# Crystal structure and Hirshfeld surface analysis of 1-[(*E*)-2-(5-chloro-2-hy­droxy­phen­yl)hydrazin-1-yl­idene]naphthalen-2(1*H*)-one

**DOI:** 10.1107/S2056989021005491

**Published:** 2021-05-28

**Authors:** Hassiba Bougueria, Souheyla Chetioui, Mohammed Abdellatif Bensegueni, Jean-Pierre Djukic, Nesrine Benarous

**Affiliations:** a Unité de Recherche de Chimie de l’Environnement et Moléculaire Structurale (URCHEMS), Département de Chimie, Université des Frères Mentouri de Constantine-1, 25000 Constantine, Algeria; b Centre Universitaire Abd El Hafid Boussouf, Mila, 43000 Mila, Algeria; c Faculté de Technologie, Université Mohamed Boudiaf M’sila, Algeria; d Laboratoire de Chimie et Systémique Organométallique (LCSOM), Institut de Chimie, Université de Strasbourg, UMR 7177, 4 rue Blaise Pascal, F-67070 Strasbourg Cedex, France

**Keywords:** azo compounds, 2-naphthols, crystal structure, Hirshfeld surface calculations

## Abstract

The title compound, C_16_H_11_ClN_2_O_2_, was obtained by diazo­tization of 2-amino-4-chloro­phenol followed by a coupling reaction with β-naphthol. There are two mol­ecules (*A* and *B*) in the asymmetric unit. The crystal structure features only one type of inter­molecular inter­action, that is strong hydrogen bonds involving the hydroxyl group. The naphthol and phenol fragments attached to the C=N—N— moiety exhibit an *s*-*trans* conformation. In addition, those fragments are almost coplanar, subtending a dihedral angle of 13.11 (2)° in mol­ecule *A* and 10.35 (2)° in mol­ecule *B*. A Hirshfeld surface analysis indicates that the most important contributions to the crystal packing are from H⋯H (32.1%), C⋯H/H⋯C (23.1%), Cl⋯H/H⋯Cl (15.2%), O⋯H/H⋯O (12.8%) and C⋯C (9%) contacts.

## Abstract 2

The mol­ecular structure of the newly synthesized dye (*E*)-1-[2-(5-chloro-2-hy­droxy­phen­yl)hydrazinyl­idene]naphthalen-2(1*H*)-one was determined by X-ray diffraction at 173 K. The asymmetric unit of the title contains two crystallographically independent mol­ecules, which adopt an *s*-*trans* conformation.

## Chemical context   

Azo compounds are one of the most frequently used compounds in organic chemistry, mainly due to their relatively simple preparation methods. They have therefore been widely used in industry, particularly as dyes for textiles (Ramugade *et al.*, 2019 ▸), printing (Benkhaya *et al.*, 2020 ▸; Choi *et al.*, 2019 ▸), cosmetics (Guerra *et al.*, 2018 ▸) and food additives (Wu *et al.*, 2019 ▸). Apart from their use as colourants, azo compounds have attracted a lot of attention from chemists as their potential applications are important in coordination chemistry (Asha & Mandal, 2018 ▸), metal–organic frameworks (MOFs) (Huang *et al.*, 2017 ▸), covalent–organic frameworks (COFs) (Chandra *et al.*, 2014 ▸) and catalysis (Choudhary *et al.*, 2017 ▸). In addition, they have found many applications in different fields such as non-linear optics (Dudek *et al.*, 2020 ▸), optical storage (Kovalchuk *et al.*, 2020 ▸), photoluminescence (He *et al.*, 2019 ▸), chemosensors (Akram *et al.*, 2020 ▸) and magnetism (Nandi *et al.*, 2021 ▸). They are used not only in physics but also in the biomedical and pharmacological fields as they can offer new therapeutic properties such as anti­viral (Chhetri *et al.*, 2021 ▸), anti­microbial (Kyei *et al.*, 2020 ▸), anti-inflammatory and anti­oxidant (Unnisa *et al.*, 2020 ▸). On the other hand, azo-naphthol derivatives form a widely studied class of azo compounds. Considerable research has been devoted to the development of new dyes prepared by the azo coupling reaction, which occurs between diazo­nium salts and 1- or 2-naphthols (Shalini Rosalyn *et al.*, 2007 ▸; Bougueria *et al.*, 2013*a* ▸; Gusev *et al.*, 2018 ▸). Following our inter­est in this area, we describe here the crystal structure of a novel azo compound derived from β-naphthol and 2-amino-4-chloro­phenol, *viz.* 1-[(*E*)-2-(5-chloro-2-hy­droxy­phen­yl)hydrazin-1-yl­idene]naphthalen-2(1*H*)-one.
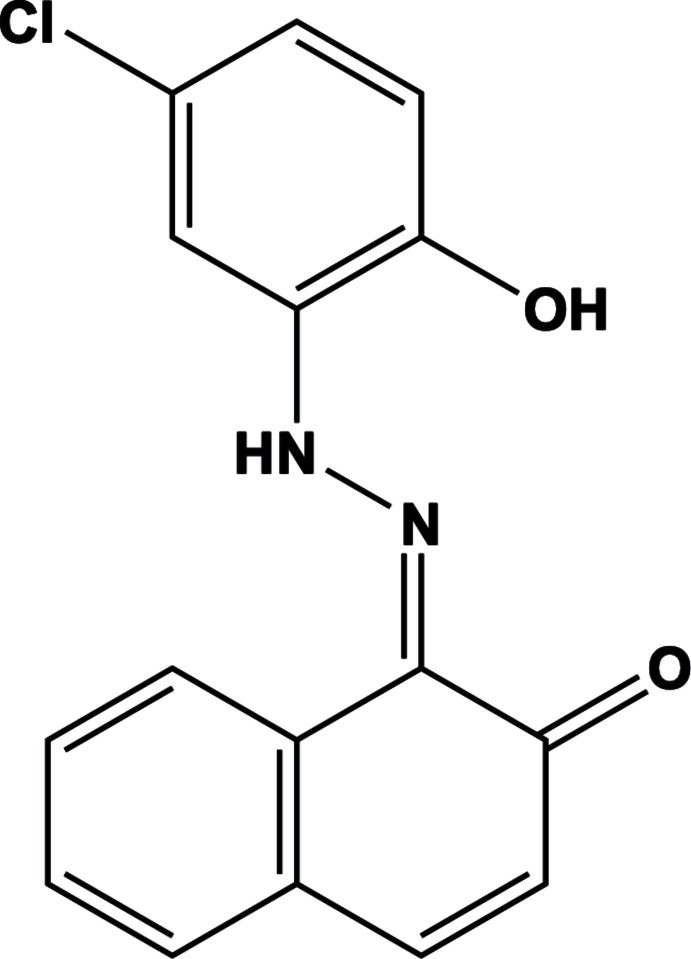


## Structural commentary   

**Figure 1:**
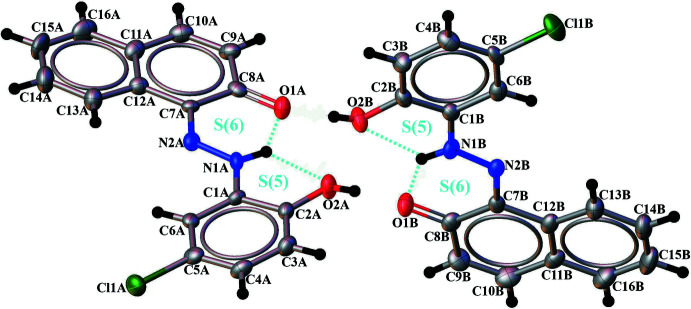
View of the two independent mol­ecules of the title compound, showing the atom-labelling scheme. Displacement ellipsoids are drawn at the 50% probability level. Intra­molecular hydrogen bonds are shown as dashed lines.

**Figure 2:**
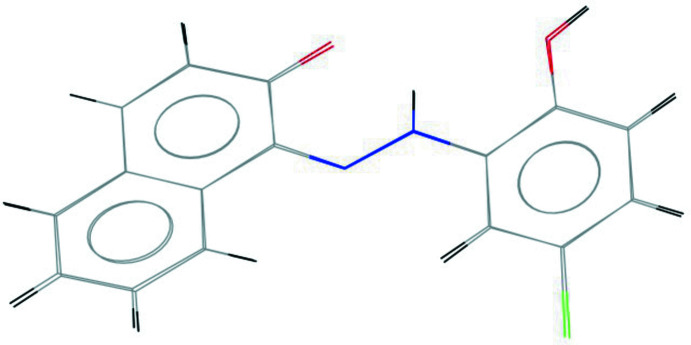
Overlay image of the two mol­ecules in the asymmetric unit of the title compound.

The asymmetric unit of title compound contains two crystallographically independent mol­ecules (*A* and *B*) in which the N1*A*—N2*A*, N1*B*—N2*B*, C8*A*—O1*A* and C8*B*—O1*B* bond lengths are 1.307 (5), 1.307 (5), 1.262 (7) and 1.271 (7) Å, respectively, which indicates that the dye compound has crystallized in its neutral hydrazo tautomeric form (Fig. 1 ▸); this is common when there is a OH group in the *ortho*-position corresponding to the azo group. Bond lengths and angles are within normal ranges and are comparable to those observed in related structures (Bougueria *et al.*, 2014 ▸; Chetioui *et al.*, 2013*a* ▸). The conformational differences between mol­ecules *A* and *B* are highlighted in an overlay diagram shown in Fig. 2 ▸. The naphthol and phenol rings attached to the hydrazo group are almost coplanar, subtending a dihedral angle of 13.11 (2)° in mol­ecule *A* and 10.35 (2)° in mol­ecule *B*, indicating significant electron delocalization within the mol­ecules. The mol­ecular structures of *A* and *B* are each stabilized by two intra­molecular N—H⋯O hydrogen bonds with *S*(6) and *S*(5) motifs and involving the hydrogen atoms from the hydrazo groups (Table 1 ▸, Fig. 1 ▸).

## Supra­molecular features   

**Figure 3:**
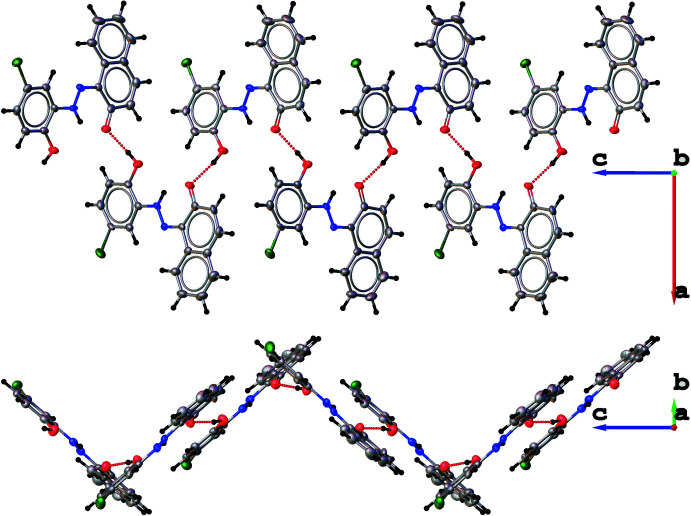
A partial packing diagram of the title compound showing a zigzag chain formation along the *c* axis.

In the crystal, the presence of hydroxyl groups leads indeed to the formation of inter­molecular O—H⋯O hydrogen bonds, generating infinite zigzag chains along the *c*-axis direction (Table 1 ▸, Fig. 3 ▸). No significant π–π stacking inter­actions were observed, despite the presence of aromatic rings in the mol­ecules.

## Analysis of the Hirshfeld surfaces   

**Figure 4:**
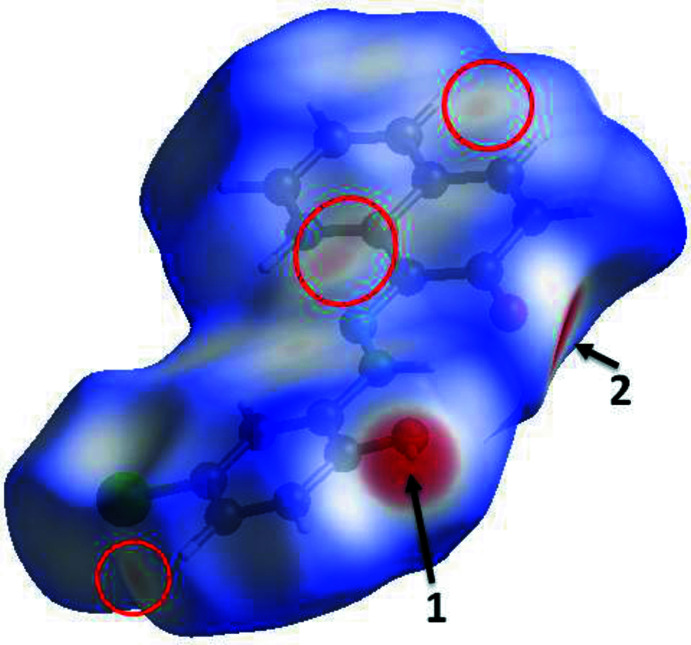
Hirshfeld surface mapped over *d*_norm_ for the title compound in the range −0.728 to +1.258 arbitrary units.

**Figure 5:**
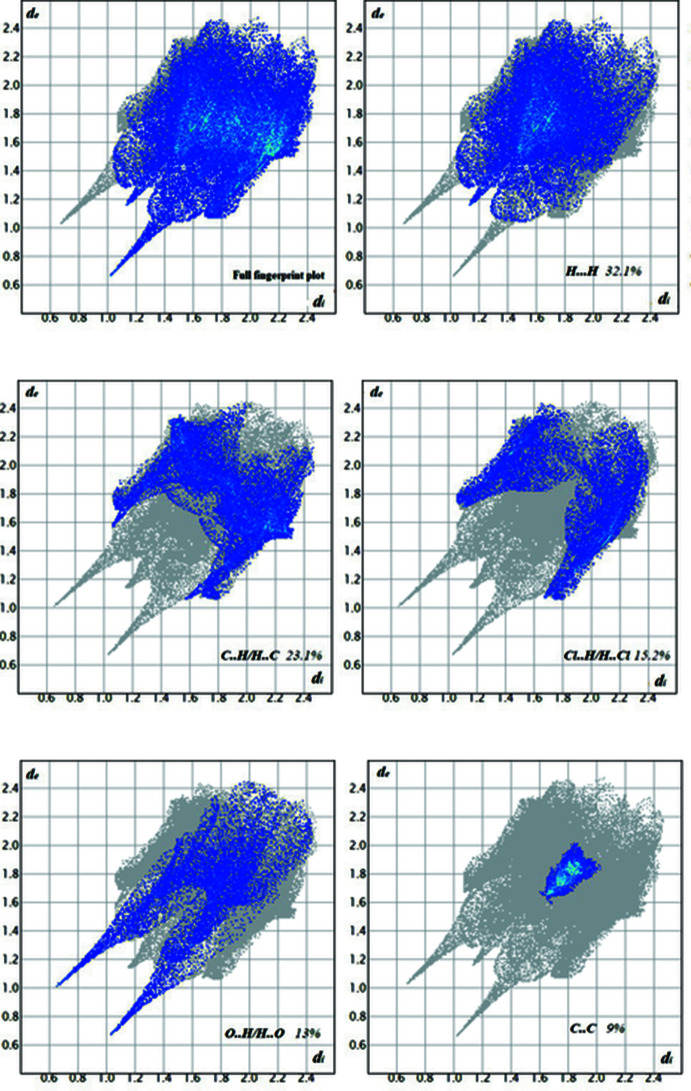
The full fingerprint plot for title compound and those delineated into H⋯H, C⋯H/H⋯C, Cl⋯H/H⋯Cl, O⋯H/H⋯O and C⋯C contacts.

**Figure 6:**
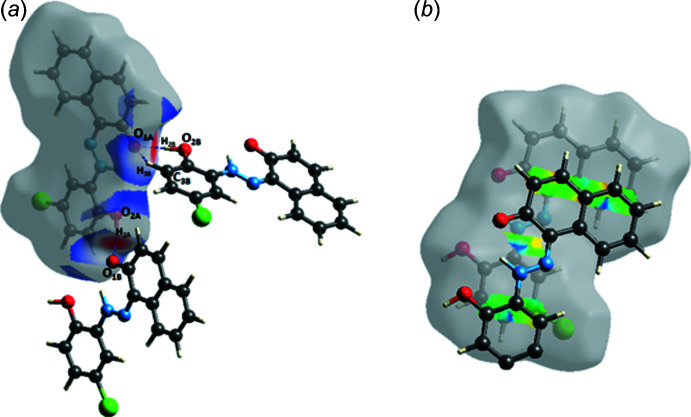
Hirshfeld surface mapped over *d*_norm_ for the title compound showing: (*a*) O⋯H/H⋯O contacts and (*b*) C⋯C contacts.

A Hirshfeld surface analysis (Spackman & Jayatilaka, 2009 ▸) was undertaken using *CrystalExplorer17* (Turner *et al.*, 2017 ▸) and the associated two-dimensional fingerprint plots (McKinnon *et al.*, 2007 ▸) were generated. The Hirshfeld (HS) surfaces of the title compound mapped over *d*_norm_ are given in Fig. 4 ▸. The normalized contact distance, *d*_norm_, varies from red to blue to white depending on the contact distances relative the sum of the van der Waals radius The intense red spots labelled 1 and 2 are related to the presence of O—H⋯O hydrogen bonds in the crystal structure. Weak contacts are highlighted by red circles. More significant contacts and their percentage contributions to the Hirshfeld surface are given in Table 2 ▸. The two-dimensional fingerprint plots are shown in Fig. 5 ▸. They reveal that the main contributions to the HS are from H⋯H (32.1%), C⋯H/H⋯C (23.1%), Cl⋯H/H⋯Cl (15.2%), O⋯H/H⋯O (12.8%, Fig. 6 ▸ *a*) and C⋯C (9%, Fig. 6 ▸ *b*) contacts.

## Database survey   

A search of the Cambridge Structural Database (CSD version 2020.3.0, update of February 2021; Groom *et al.*, 2016 ▸) revealed that several examples of structurally similar azo-naphthol compounds were prepared using different aromatic primary amine, *viz*. (*E*)-1-[2-(2-cyano­phen­yl)diazen-2-ium-1-yl]naphthalen-2-olate (Bougueria *et al.*, 2013*b* ▸), (*E*)-1-(4-fluoro­phen­yl)-2-(2-oxidonaphthalen-1-yl)diazenium (Bou­gueria *et al.*, 2017 ▸), 4-[(2-aphthalen-1-yl)diazen­yl]benzene­sulfonamide (Benosmane *et al.*, 2012 ▸), 1-(3-acetyl­phen­yl)-2-(2-oxidonaphthalen-1-yl)diazen-1-ium (Bougueria *et al.*, 2013*c* ▸), (*E*)-1-(3-chloro­phen­yl)-2-(2-oxidonaphthalen-1-yl)diazen-1-ium (Benosmane *et al.*, 2013 ▸), (*E*)-1-[(2,4,6-tri­bromo­phen­yl)diazen­yl]naphthalen-2-ol (Chetioui *et al.*, 2013*b* ▸).

## Synthesis and crystallization   

The title compound was synthesized according to a reported method (Wang *et al.*, 2003 ▸). A solution of hydro­chloric acid (12 mmol, in 6 mL of water) was added to 2-amino-4-chloro­phenol (12 mmol) at 273 K. Sodium nitrite solution (24 mmol, in 8 mL of water) was added dropwise to the cooled mixture and stirred for 20 min. To the formed diazo­nium chloride was added dropwise an aqueous solution of 2-naphthol (12 mmol in 100 mL of water) containing hydroxide sodium (16 mL). The produced mixture was allowed to stir for 1 h at 278 K. The resulting red precipitate was filtered and washed with water several times. The crude azo dye was recrystallized from hot ethanol giving a pure azo dye in a good yield (80.0%). Single crystals suitable for X-ray analysis, were obtained by dissolving the compound in a minimum amount of THF/H_2_O (1/1 *v*/v) at room temperature. To confirm the formula of the compound, an elementary analysis was carried out: calculated for C_16_H_11_N_2_OCl, C 64.33%, N 9.38%, H 3.71%, found C 64.41%, N 8.45%, H 3.70%. The IR spectra (KBr pellet) were recorded using a Shimadzu FTIR 8000 series Fourier transform spectrometer in the range 4000 to 400 cm^−1^. IR (cm^−1^): ν(C=O): 1596.91, ν(C=C): 1500, ν(C=N): 1490.43, ν(C—Cl): 745.10, ν (C—C): 1400, ν(C—H): 2921.31. NMR spectra of CDCl_3_ solutions were recorded on a Bruker Advance 400 spectrometer at 400 MHz. ^1^H NMR δ (ppm) 7.031–8.209 (9H, aromatic group protons), 12.414 (singlet, 1H, OH phenol) and 14.38 (singlet, 1H, N—H⋯O). ^13^C NMR δ (ppm) 156.86 (C=O), 150.49 (C=N), (109.49–136.92) (C—H).

## Refinement details   

Crystal data, data collection and structure refinement details are summarized in Table 3 ▸. The hydrogen atoms of hydroxyl and hydrazo groups were localized in a difference-Fourier map and refined with O—H = 0.84 (1) Å and N—H = 0.88 (1) Å, respectively, and with *U*_iso_(H) set to 1.5*U*_eq_(O) or 1.2*U*_eq_(N). The other hydrogen atoms were placed in calculated positions with C—H = 0.93 Å and refined using a riding model with fixed isotropic displacement parameters [*U*_iso_(H) = 1.2*U*_eq_(C)].

## Supplementary Material

Crystal structure: contains datablock(s) I. DOI: 10.1107/S2056989021005491/zn2007sup1.cif

Structure factors: contains datablock(s) I. DOI: 10.1107/S2056989021005491/zn2007Isup2.hkl

Supporting information file. DOI: 10.1107/S2056989021005491/zn2007Isup3.cml

CCDC reference: 2085853

Additional supporting information:  crystallographic information; 3D view; checkCIF report

## Figures and Tables

**Table 1:** Hydrogen-bond geometry (Å, °)

*D*—H⋯*A*	*D*—H	H⋯*A*	*D*⋯*A*	*D*—H⋯*A*
N1*A*—H1*A*⋯O1*A*	0.89 (4)	1.86 (5)	2.550 (7)	133 (4)
N1*A*—H1*A*⋯O2*A*	0.89 (4)	2.35 (5)	2.666 (6)	101 (4)
N1*B*—H1*B*⋯O1*B*	0.88 (4)	1.91 (5)	2.584 (6)	132 (4)
N1*B*—H1*B*⋯O2*B*	0.88 (4)	2.34 (4)	2.673 (6)	103 (4)
O2*A*—H2*A*⋯O1*B* ^i^	0.84 (5)	1.85 (5)	2.674 (6)	168 (5)
O2*B*—H2*B*⋯O1*A*	0.85 (6)	1.82 (6)	2.656 (7)	173 (6)

Symmetry code: (i) x, -y+1, z+{\script{1\over 2}}.

**Table 2:** Percentage contributions of various contacts to the Hirshfeld surface

Contact	Percentage contribution
H⋯H	32.1
C⋯H/H⋯C	23.1
Cl⋯H/H⋯Cl	15.2
O⋯H/H⋯O	12.8
C⋯C	9
Cl⋯C/C⋯Cl	2.2
O⋯O	0.9
C⋯O/O⋯C	1.2

**Table 3:** Experimental details

Crystal data	
Chemical formula	C_16_H_11_ClN_2_O_2_
*M*_r_	298.72
Crystal system, space group	Monoclinic, *C* *c*
Temperature (K)	173
*a*, *b*, *c* (Å)	32.830 (4), 4.4049 (5), 18.844 (2)
β (°)	90.130 (3)
*V* (Å^3^)	2725.1 (6)
*Z*	8
Radiation type	Mo *K*α
μ (mm^−1^)	0.29
Crystal size (mm)	0.3 × 0.2 × 0.06

Data collection	
Diffractometer	Bruker APEXII CCD
Absorption correction	Multi-scan (*SADABS*; Sheldrick, 2002 ▸)
*T*_min_, *T*_max_	0.610, 0.746
No. of measured, independent and observed [*I* > 2σ(*I*)] reflections	13940, 6168, 4497
*R*_int_	0.063
(sin θ/λ)_max_ (Å^−1^)	0.660

Refinement	
*R*[*F* ^2^ > 2σ(*F* ^2^)], *wR*(*F* ^2^), *S*	0.046, 0.082, 0.97
No. of reflections	6168
No. of parameters	392
No. of restraints	6
H-atom treatment	H atoms treated by a mixture of independent and constrained refinement
Δρ_max_, Δρ_min_ (e Å^−3^)	0.34, −0.27
Absolute structure	Flack *x* determined using 1605 quotients [(*I* ^+^)−(*I* ^−^)]/[(*I* ^+^)+(*I* ^−^)] (Parsons *et al.* (2013 ▸)
Absolute structure parameter	−0.02 (3)

Computer programs: *APEX2* and *SAINT* (Bruker, 2014 ▸), *SHELXT2018/3* (Sheldrick, 2015*a* ▸), *SHELXL2018/3* (Sheldrick, 2015*b* ▸) and *OLEX2* (Dolomanov *et al.*, 2009 ▸).

## supplementary crystallographic information

### Crystal data

**Table:**

C_16_H_11_ClN_2_O_2_	*F*(000) = 1232
*M**_r_* = 298.72	*D*_x_ = 1.456 Mg m^−^^3^
Monoclinic, *C**c*	Mo *K*α radiation, λ = 0.71073 Å
*a* = 32.830 (4) Å	Cell parameters from 13940 reflections
*b* = 4.4049 (5) Å	θ = 1.6–28.0°
*c* = 18.844 (2) Å	µ = 0.29 mm^−^^1^
β = 90.130 (3)°	*T* = 173 K
*V* = 2725.1 (6) Å^3^	Plate, red
*Z* = 8	0.3 × 0.2 × 0.06 mm

### Data collection

**Table:**

Bruker APEXII CCD diffractometer	4497 reflections with *I* > 2σ(*I*)
φ and ω scans	*R*_int_ = 0.063
Absorption correction: multi-scan (SADABS; Sheldrick, 2002)	θ_max_ = 28.0°, θ_min_ = 1.6°
*T*_min_ = 0.610, *T*_max_ = 0.746	*h* = −42→42
13940 measured reflections	*k* = −5→5
6168 independent reflections	*l* = −23→24

### Refinement

**Table:**

Refinement on *F*^2^	Hydrogen site location: mixed
Least-squares matrix: full	H atoms treated by a mixture of independent and constrained refinement
*R*[*F*^2^ > 2σ(*F*^2^)] = 0.046	*w* = 1/[σ^2^(*F*_o_^2^) + (0.0325*P*)^2^] where *P* = (*F*_o_^2^ + 2*F*_c_^2^)/3
*wR*(*F*^2^) = 0.082	(Δ/σ)_max_ < 0.001
*S* = 0.97	Δρ_max_ = 0.34 e Å^−^^3^
6168 reflections	Δρ_min_ = −0.27 e Å^−^^3^
392 parameters	Absolute structure: Flack *x* determined using 1605 quotients [(*I*^+^)-(*I*^-^)]/[(*I*^+^)+(*I*^-^)] (Parsons *et al.* (2013)
6 restraints	Absolute structure parameter: −0.02 (3)

### Special details

**Table:**

Geometry. All esds (except the esd in the dihedral angle between two l.s. planes) are estimated using the full covariance matrix. The cell esds are taken into account individually in the estimation of esds in distances, angles and torsion angles; correlations between esds in cell parameters are only used when they are defined by crystal symmetry. An approximate (isotropic) treatment of cell esds is used for estimating esds involving l.s. planes.
Refinement. Refined as a 2-component twin.

### Fractional atomic coordinates and isotropic or equivalent isotropic displacement parameters (Å^2^)

**Table:**

	*x*	*y*	*z*	*U*_iso_*/*U*_eq_	
Cl1A	0.33793 (5)	−0.0030 (4)	0.79791 (9)	0.0327 (5)	
Cl1B	0.66132 (5)	1.4840 (4)	0.55207 (9)	0.0397 (5)	
O1B	0.54584 (14)	0.4608 (10)	0.2885 (2)	0.0302 (12)	
O1A	0.45074 (14)	1.0202 (9)	0.5356 (3)	0.0293 (12)	
O2A	0.48688 (13)	0.5655 (11)	0.6906 (2)	0.0325 (11)	
H2A	0.5072 (14)	0.541 (14)	0.717 (3)	0.049*	
N1A	0.41552 (14)	0.7116 (10)	0.6321 (2)	0.0214 (11)	
H1A	0.4385 (10)	0.795 (11)	0.616 (3)	0.026*	
N2A	0.38083 (14)	0.8261 (10)	0.6109 (2)	0.0238 (11)	
O2B	0.51014 (13)	0.9509 (10)	0.4415 (2)	0.0325 (11)	
H2B	0.4928 (17)	0.978 (14)	0.474 (3)	0.049*	
C3A	0.45484 (18)	0.2251 (13)	0.7736 (3)	0.0275 (14)	
H3A	0.480248	0.176258	0.794936	0.033*	
C4A	0.41959 (19)	0.0909 (14)	0.7992 (3)	0.0264 (14)	
H4A	0.420616	−0.050199	0.837289	0.032*	
N1B	0.58132 (14)	0.7832 (11)	0.3851 (2)	0.0225 (11)	
H1B	0.5580 (9)	0.721 (11)	0.367 (3)	0.027*	
C5A	0.38280 (18)	0.1682 (13)	0.7677 (3)	0.0263 (14)	
N2B	0.61607 (14)	0.6715 (10)	0.3637 (2)	0.0202 (10)	
C11A	0.33655 (18)	1.3521 (14)	0.4820 (3)	0.0285 (14)	
C6A	0.38021 (16)	0.3698 (12)	0.7126 (3)	0.0210 (13)	
H6A	0.354633	0.417884	0.691739	0.025*	
C1A	0.41578 (19)	0.5025 (12)	0.6878 (3)	0.0198 (14)	
C12A	0.33986 (18)	1.1471 (13)	0.5398 (3)	0.0243 (13)	
C2A	0.45390 (17)	0.4265 (14)	0.7182 (3)	0.0231 (13)	
C9A	0.41010 (19)	1.3450 (13)	0.4640 (3)	0.0282 (14)	
H9A	0.433088	1.416994	0.438520	0.034*	
C7A	0.3799 (2)	1.0286 (13)	0.5580 (3)	0.0180 (15)	
C1B	0.58115 (19)	0.9956 (12)	0.4404 (3)	0.0215 (14)	
C8A	0.41619 (17)	1.1280 (12)	0.5204 (3)	0.0221 (12)	
C11B	0.66111 (19)	0.1491 (14)	0.2370 (3)	0.0270 (13)	
C10A	0.3725 (2)	1.4493 (14)	0.4463 (3)	0.0293 (15)	
H10A	0.370064	1.592201	0.408711	0.035*	
C7B	0.6165 (2)	0.4648 (13)	0.3122 (3)	0.0204 (15)	
C6B	0.61767 (17)	1.1186 (13)	0.4666 (3)	0.0252 (14)	
H6B	0.643110	1.060965	0.446860	0.030*	
C9B	0.58710 (19)	0.1460 (14)	0.2176 (3)	0.0302 (15)	
H9B	0.564299	0.071472	0.191826	0.036*	
C8B	0.58115 (17)	0.3618 (13)	0.2735 (3)	0.0245 (13)	
C2B	0.54441 (18)	1.0792 (14)	0.4703 (3)	0.0238 (13)	
C3B	0.54349 (18)	1.2819 (13)	0.5257 (3)	0.0271 (14)	
H3B	0.518167	1.337103	0.546279	0.033*	
C4B	0.5795 (2)	1.4071 (15)	0.5518 (3)	0.0302 (15)	
H4B	0.579068	1.548100	0.589890	0.036*	
C13B	0.69225 (19)	0.4422 (14)	0.3309 (4)	0.0320 (15)	
H13B	0.690062	0.577789	0.369884	0.038*	
C14A	0.2675 (2)	1.1832 (16)	0.5571 (4)	0.0446 (18)	
H14A	0.243793	1.130825	0.583282	0.054*	
C5B	0.61592 (16)	1.3217 (13)	0.5209 (3)	0.0226 (13)	
C15A	0.2637 (2)	1.3754 (17)	0.4983 (4)	0.0453 (19)	
H15A	0.237675	1.444671	0.483537	0.054*	
C13A	0.30454 (19)	1.0684 (15)	0.5779 (4)	0.0318 (15)	
H13A	0.306332	0.936823	0.617770	0.038*	
C10B	0.6250 (2)	0.0474 (14)	0.2012 (4)	0.0352 (17)	
H10B	0.627872	−0.096770	0.164073	0.042*	
C12B	0.65732 (17)	0.3528 (14)	0.2939 (3)	0.0245 (13)	
C16A	0.2976 (2)	1.4619 (15)	0.4627 (4)	0.0407 (19)	
H16A	0.295169	1.598838	0.423940	0.049*	
C14B	0.72996 (19)	0.3336 (16)	0.3108 (3)	0.0366 (16)	
H14B	0.753507	0.392677	0.336718	0.044*	
C15B	0.7339 (2)	0.1396 (18)	0.2533 (4)	0.0439 (18)	
H15B	0.760132	0.072960	0.238667	0.053*	
C16B	0.6999 (2)	0.0451 (16)	0.2179 (4)	0.0404 (19)	
H16B	0.702620	−0.093672	0.179603	0.048*	

### Atomic displacement parameters (Å^2^)

**Table:**

	*U*^11^	*U*^22^	*U*^33^	*U*^12^	*U*^13^	*U*^23^
Cl1A	0.0279 (10)	0.0326 (10)	0.0379 (12)	−0.0041 (7)	0.0076 (7)	0.0030 (7)
Cl1B	0.0341 (11)	0.0507 (13)	0.0343 (12)	−0.0168 (8)	−0.0114 (7)	−0.0005 (8)
O1B	0.019 (2)	0.040 (3)	0.032 (3)	0.0019 (18)	−0.007 (2)	−0.007 (2)
O1A	0.019 (2)	0.037 (3)	0.033 (3)	0.0005 (19)	−0.001 (2)	0.000 (2)
O2A	0.017 (2)	0.043 (3)	0.038 (3)	−0.001 (2)	−0.005 (2)	0.008 (2)
N1A	0.014 (3)	0.023 (3)	0.027 (3)	−0.002 (2)	0.000 (2)	−0.004 (2)
N2A	0.020 (2)	0.025 (3)	0.026 (3)	0.000 (2)	−0.007 (2)	−0.004 (2)
O2B	0.020 (2)	0.045 (3)	0.032 (3)	−0.008 (2)	0.0028 (19)	−0.010 (2)
C3A	0.025 (3)	0.030 (4)	0.027 (3)	0.006 (3)	−0.005 (2)	−0.001 (3)
C4A	0.033 (4)	0.025 (3)	0.021 (3)	0.009 (3)	0.001 (3)	0.004 (3)
N1B	0.018 (3)	0.029 (3)	0.020 (3)	0.003 (2)	−0.005 (2)	0.001 (2)
C5A	0.029 (3)	0.022 (3)	0.028 (3)	0.004 (3)	0.007 (3)	−0.003 (3)
N2B	0.019 (2)	0.024 (3)	0.017 (2)	0.002 (2)	−0.0003 (19)	0.004 (2)
C11A	0.029 (3)	0.031 (3)	0.026 (3)	0.005 (3)	−0.008 (3)	−0.006 (3)
C6A	0.021 (3)	0.017 (3)	0.025 (3)	−0.001 (2)	−0.003 (2)	−0.006 (3)
C1A	0.022 (3)	0.017 (3)	0.020 (3)	0.005 (2)	−0.003 (3)	0.000 (2)
C12A	0.027 (3)	0.022 (3)	0.023 (3)	0.000 (3)	−0.004 (2)	−0.001 (3)
C2A	0.019 (3)	0.023 (3)	0.028 (3)	0.001 (3)	−0.002 (2)	−0.004 (3)
C9A	0.035 (4)	0.020 (3)	0.030 (4)	0.001 (3)	0.007 (3)	0.001 (3)
C7A	0.023 (3)	0.012 (3)	0.019 (3)	0.000 (2)	−0.002 (2)	−0.001 (3)
C1B	0.022 (4)	0.022 (4)	0.021 (3)	−0.001 (3)	−0.003 (3)	0.003 (2)
C8A	0.027 (3)	0.018 (3)	0.021 (3)	−0.005 (3)	0.000 (2)	−0.006 (3)
C11B	0.030 (3)	0.026 (3)	0.025 (3)	0.007 (3)	0.002 (2)	0.004 (3)
C10A	0.041 (4)	0.024 (3)	0.023 (3)	0.004 (3)	−0.002 (3)	0.000 (3)
C7B	0.021 (3)	0.023 (4)	0.018 (3)	−0.001 (3)	−0.003 (2)	0.007 (3)
C6B	0.023 (3)	0.031 (3)	0.022 (3)	0.001 (3)	−0.002 (2)	0.006 (3)
C9B	0.035 (4)	0.038 (4)	0.018 (3)	−0.003 (3)	−0.008 (3)	−0.005 (3)
C8B	0.027 (3)	0.027 (3)	0.020 (3)	0.004 (3)	−0.002 (2)	0.005 (3)
C2B	0.021 (3)	0.027 (3)	0.023 (3)	−0.005 (3)	−0.003 (2)	0.003 (3)
C3B	0.025 (3)	0.030 (4)	0.026 (3)	0.000 (3)	0.005 (2)	−0.004 (3)
C4B	0.037 (4)	0.033 (3)	0.021 (3)	−0.008 (3)	−0.003 (3)	−0.002 (3)
C13B	0.024 (3)	0.038 (4)	0.034 (4)	0.000 (3)	−0.001 (3)	0.001 (3)
C14A	0.023 (3)	0.053 (5)	0.058 (5)	0.000 (3)	−0.004 (3)	−0.004 (4)
C5B	0.020 (3)	0.026 (3)	0.022 (3)	−0.008 (2)	−0.009 (2)	0.008 (3)
C15A	0.026 (4)	0.049 (5)	0.061 (5)	0.012 (3)	−0.017 (3)	0.004 (4)
C13A	0.024 (3)	0.037 (4)	0.035 (4)	0.001 (3)	−0.001 (3)	0.000 (3)
C10B	0.049 (5)	0.035 (4)	0.022 (3)	0.005 (3)	−0.001 (3)	−0.008 (3)
C12B	0.023 (3)	0.028 (3)	0.022 (3)	0.004 (3)	0.002 (3)	0.008 (3)
C16A	0.044 (5)	0.041 (4)	0.037 (4)	0.009 (3)	−0.019 (3)	0.001 (3)
C14B	0.021 (3)	0.048 (4)	0.041 (4)	0.002 (3)	−0.004 (3)	0.007 (4)
C15B	0.026 (4)	0.055 (5)	0.051 (5)	0.012 (3)	0.008 (3)	0.010 (4)
C16B	0.041 (4)	0.040 (4)	0.040 (4)	0.011 (3)	0.008 (3)	−0.002 (3)

### Geometric parameters (Å, º)

**Table:**

Cl1A—C5A	1.751 (6)	C1B—C6B	1.405 (8)
Cl1B—C5B	1.753 (6)	C1B—C2B	1.383 (8)
O1B—C8B	1.271 (7)	C11B—C10B	1.433 (9)
O1A—C8A	1.262 (7)	C11B—C12B	1.405 (8)
O2A—H2A	0.834 (14)	C11B—C16B	1.402 (9)
O2A—C2A	1.350 (7)	C10A—H10A	0.9500
N1A—H1A	0.891 (14)	C7B—C8B	1.443 (9)
N1A—N2A	1.307 (5)	C7B—C12B	1.469 (9)
N1A—C1A	1.397 (7)	C6B—H6B	0.9500
N2A—C7A	1.338 (7)	C6B—C5B	1.360 (8)
O2B—H2B	0.842 (14)	C9B—H9B	0.9500
O2B—C2B	1.370 (7)	C9B—C8B	1.433 (8)
C3A—H3A	0.9500	C9B—C10B	1.355 (9)
C3A—C4A	1.388 (8)	C2B—C3B	1.373 (8)
C3A—C2A	1.370 (8)	C3B—H3B	0.9500
C4A—H4A	0.9500	C3B—C4B	1.393 (8)
C4A—C5A	1.387 (8)	C4B—H4B	0.9500
N1B—H1B	0.885 (14)	C4B—C5B	1.382 (8)
N1B—N2B	1.307 (5)	C13B—H13B	0.9500
N1B—C1B	1.400 (7)	C13B—C12B	1.397 (8)
C5A—C6A	1.369 (8)	C13B—C14B	1.381 (9)
N2B—C7B	1.329 (7)	C14A—H14A	0.9500
C11A—C12A	1.420 (8)	C14A—C15A	1.399 (10)
C11A—C10A	1.426 (9)	C14A—C13A	1.374 (9)
C11A—C16A	1.413 (9)	C15A—H15A	0.9500
C6A—H6A	0.9500	C15A—C16A	1.356 (10)
C6A—C1A	1.387 (8)	C13A—H13A	0.9500
C1A—C2A	1.415 (8)	C10B—H10B	0.9500
C12A—C7A	1.454 (9)	C16A—H16A	0.9500
C12A—C13A	1.409 (9)	C14B—H14B	0.9500
C9A—H9A	0.9500	C14B—C15B	1.386 (10)
C9A—C8A	1.443 (8)	C15B—H15B	0.9500
C9A—C10A	1.359 (8)	C15B—C16B	1.364 (10)
C7A—C8A	1.455 (9)	C16B—H16B	0.9500
			
C2A—O2A—H2A	111 (5)	N2B—C7B—C12B	114.4 (6)
N2A—N1A—H1A	119 (4)	C8B—C7B—C12B	120.6 (5)
N2A—N1A—C1A	119.2 (5)	C1B—C6B—H6B	120.6
C1A—N1A—H1A	121 (4)	C5B—C6B—C1B	118.8 (5)
N1A—N2A—C7A	120.2 (5)	C5B—C6B—H6B	120.6
C2B—O2B—H2B	102 (5)	C8B—C9B—H9B	119.8
C4A—C3A—H3A	119.2	C10B—C9B—H9B	119.8
C2A—C3A—H3A	119.2	C10B—C9B—C8B	120.5 (6)
C2A—C3A—C4A	121.6 (6)	O1B—C8B—C7B	120.9 (6)
C3A—C4A—H4A	120.9	O1B—C8B—C9B	121.2 (5)
C5A—C4A—C3A	118.2 (6)	C9B—C8B—C7B	118.0 (5)
C5A—C4A—H4A	120.9	O2B—C2B—C1B	116.4 (5)
N2B—N1B—H1B	121 (4)	O2B—C2B—C3B	123.3 (5)
N2B—N1B—C1B	119.1 (5)	C3B—C2B—C1B	120.3 (5)
C1B—N1B—H1B	120 (4)	C2B—C3B—H3B	119.8
C4A—C5A—Cl1A	119.1 (5)	C2B—C3B—C4B	120.3 (5)
C6A—C5A—Cl1A	118.4 (5)	C4B—C3B—H3B	119.8
C6A—C5A—C4A	122.5 (5)	C3B—C4B—H4B	120.7
N1B—N2B—C7B	119.6 (5)	C5B—C4B—C3B	118.6 (6)
C12A—C11A—C10A	119.4 (5)	C5B—C4B—H4B	120.7
C16A—C11A—C12A	118.9 (6)	C12B—C13B—H13B	120.0
C16A—C11A—C10A	121.6 (6)	C14B—C13B—H13B	120.0
C5A—C6A—H6A	120.7	C14B—C13B—C12B	120.1 (6)
C5A—C6A—C1A	118.5 (5)	C15A—C14A—H14A	119.2
C1A—C6A—H6A	120.7	C13A—C14A—H14A	119.2
N1A—C1A—C2A	117.6 (5)	C13A—C14A—C15A	121.6 (7)
C6A—C1A—N1A	121.8 (5)	C6B—C5B—Cl1B	118.9 (5)
C6A—C1A—C2A	120.6 (5)	C6B—C5B—C4B	122.2 (5)
C11A—C12A—C7A	118.5 (5)	C4B—C5B—Cl1B	118.9 (5)
C13A—C12A—C11A	119.1 (6)	C14A—C15A—H15A	120.3
C13A—C12A—C7A	122.4 (5)	C16A—C15A—C14A	119.5 (6)
O2A—C2A—C3A	124.8 (5)	C16A—C15A—H15A	120.3
O2A—C2A—C1A	116.5 (5)	C12A—C13A—H13A	120.2
C3A—C2A—C1A	118.7 (5)	C14A—C13A—C12A	119.6 (7)
C8A—C9A—H9A	119.0	C14A—C13A—H13A	120.2
C10A—C9A—H9A	119.0	C11B—C10B—H10B	118.2
C10A—C9A—C8A	122.0 (6)	C9B—C10B—C11B	123.5 (6)
N2A—C7A—C12A	115.7 (6)	C9B—C10B—H10B	118.2
N2A—C7A—C8A	123.1 (6)	C11B—C12B—C7B	118.5 (5)
C12A—C7A—C8A	121.2 (5)	C13B—C12B—C11B	119.1 (5)
N1B—C1B—C6B	121.0 (5)	C13B—C12B—C7B	122.4 (6)
C2B—C1B—N1B	119.1 (5)	C11A—C16A—H16A	119.4
C2B—C1B—C6B	119.9 (5)	C15A—C16A—C11A	121.2 (7)
O1A—C8A—C9A	122.6 (5)	C15A—C16A—H16A	119.4
O1A—C8A—C7A	120.9 (6)	C13B—C14B—H14B	119.5
C9A—C8A—C7A	116.5 (5)	C13B—C14B—C15B	120.9 (6)
C12B—C11B—C10B	119.0 (5)	C15B—C14B—H14B	119.5
C16B—C11B—C10B	121.9 (6)	C14B—C15B—H15B	120.3
C16B—C11B—C12B	119.1 (6)	C16B—C15B—C14B	119.5 (6)
C11A—C10A—H10A	118.8	C16B—C15B—H15B	120.3
C9A—C10A—C11A	122.3 (6)	C11B—C16B—H16B	119.4
C9A—C10A—H10A	118.8	C15B—C16B—C11B	121.2 (7)
N2B—C7B—C8B	125.0 (6)	C15B—C16B—H16B	119.4
			
Cl1A—C5A—C6A—C1A	−179.1 (4)	C1B—C6B—C5B—C4B	1.0 (8)
N1A—N2A—C7A—C12A	−178.5 (5)	C1B—C2B—C3B—C4B	0.6 (9)
N1A—N2A—C7A—C8A	1.3 (8)	C8A—C9A—C10A—C11A	0.0 (10)
N1A—C1A—C2A—O2A	0.4 (8)	C10A—C11A—C12A—C7A	−3.7 (8)
N1A—C1A—C2A—C3A	179.1 (5)	C10A—C11A—C12A—C13A	175.7 (6)
N2A—N1A—C1A—C6A	12.3 (8)	C10A—C11A—C16A—C15A	−177.9 (6)
N2A—N1A—C1A—C2A	−168.4 (5)	C10A—C9A—C8A—O1A	177.1 (6)
N2A—C7A—C8A—O1A	1.4 (9)	C10A—C9A—C8A—C7A	−0.6 (8)
N2A—C7A—C8A—C9A	179.3 (5)	C6B—C1B—C2B—O2B	179.6 (5)
O2B—C2B—C3B—C4B	−179.1 (6)	C6B—C1B—C2B—C3B	−0.1 (9)
C3A—C4A—C5A—Cl1A	178.9 (4)	C8B—C7B—C12B—C11B	1.5 (8)
C3A—C4A—C5A—C6A	0.2 (9)	C8B—C7B—C12B—C13B	−178.8 (6)
C4A—C3A—C2A—O2A	180.0 (6)	C8B—C9B—C10B—C11B	−0.4 (10)
C4A—C3A—C2A—C1A	1.3 (9)	C2B—C1B—C6B—C5B	−0.7 (8)
C4A—C5A—C6A—C1A	−0.4 (8)	C2B—C3B—C4B—C5B	−0.3 (9)
N1B—N2B—C7B—C8B	4.1 (9)	C3B—C4B—C5B—Cl1B	178.6 (5)
N1B—N2B—C7B—C12B	−178.9 (5)	C3B—C4B—C5B—C6B	−0.6 (9)
N1B—C1B—C6B—C5B	−179.3 (5)	C13B—C14B—C15B—C16B	−2.7 (11)
N1B—C1B—C2B—O2B	−1.7 (8)	C14A—C15A—C16A—C11A	2.5 (11)
N1B—C1B—C2B—C3B	178.6 (5)	C15A—C14A—C13A—C12A	0.6 (11)
C5A—C6A—C1A—N1A	−179.6 (5)	C13A—C12A—C7A—N2A	3.6 (8)
C5A—C6A—C1A—C2A	1.1 (8)	C13A—C12A—C7A—C8A	−176.3 (6)
N2B—N1B—C1B—C6B	8.3 (8)	C13A—C14A—C15A—C16A	−2.9 (11)
N2B—N1B—C1B—C2B	−170.3 (5)	C10B—C11B—C12B—C7B	−3.0 (9)
N2B—C7B—C8B—O1B	−2.2 (9)	C10B—C11B—C12B—C13B	177.2 (6)
N2B—C7B—C8B—C9B	177.5 (5)	C10B—C11B—C16B—C15B	−178.7 (6)
N2B—C7B—C12B—C11B	−175.8 (5)	C10B—C9B—C8B—O1B	178.6 (6)
N2B—C7B—C12B—C13B	4.0 (8)	C10B—C9B—C8B—C7B	−1.2 (8)
C11A—C12A—C7A—N2A	−177.1 (5)	C12B—C11B—C10B—C9B	2.6 (10)
C11A—C12A—C7A—C8A	3.1 (8)	C12B—C11B—C16B—C15B	−0.4 (10)
C11A—C12A—C13A—C14A	2.1 (9)	C12B—C7B—C8B—O1B	−179.1 (5)
C6A—C1A—C2A—O2A	179.7 (5)	C12B—C7B—C8B—C9B	0.6 (8)
C6A—C1A—C2A—C3A	−1.5 (9)	C12B—C13B—C14B—C15B	1.1 (10)
C1A—N1A—N2A—C7A	179.4 (5)	C16A—C11A—C12A—C7A	178.2 (6)
C12A—C11A—C10A—C9A	2.3 (9)	C16A—C11A—C12A—C13A	−2.4 (9)
C12A—C11A—C16A—C15A	0.1 (10)	C16A—C11A—C10A—C9A	−179.7 (6)
C12A—C7A—C8A—O1A	−178.8 (5)	C14B—C13B—C12B—C11B	0.8 (9)
C12A—C7A—C8A—C9A	−0.9 (8)	C14B—C13B—C12B—C7B	−179.0 (6)
C2A—C3A—C4A—C5A	−0.7 (9)	C14B—C15B—C16B—C11B	2.3 (11)
C7A—C12A—C13A—C14A	−178.6 (6)	C16B—C11B—C10B—C9B	−179.1 (6)
C1B—N1B—N2B—C7B	178.5 (5)	C16B—C11B—C12B—C7B	178.7 (6)
C1B—C6B—C5B—Cl1B	−178.2 (4)	C16B—C11B—C12B—C13B	−1.1 (9)

### Hydrogen-bond geometry (Å, º)

**Table:**

*D*—H···*A*	*D*—H	H···*A*	*D*···*A*	*D*—H···*A*
N1*A*—H1*A*···O1*A*	0.89 (4)	1.86 (5)	2.550 (7)	133 (4)
N1*A*—H1*A*···O2*A*	0.89 (4)	2.35 (5)	2.666 (6)	101 (4)
N1*B*—H1*B*···O1*B*	0.88 (4)	1.91 (5)	2.584 (6)	132 (4)
N1*B*—H1*B*···O2*B*	0.88 (4)	2.34 (4)	2.673 (6)	103 (4)
O2*A*—H2*A*···O1*B*^i^	0.84 (5)	1.85 (5)	2.674 (6)	168 (5)
O2*B*—H2*B*···O1*A*	0.85 (6)	1.82 (6)	2.656 (7)	173 (6)

Symmetry code: (i) *x*, −*y*+1, *z*+1/2.
